# Quantitative Genetics Model as the Unifying Model for Defining Genomic Relationship and Inbreeding Coefficient

**DOI:** 10.1371/journal.pone.0114484

**Published:** 2014-12-17

**Authors:** Chunkao Wang, Yang Da

**Affiliations:** Department of Animal Science, University of Minnesota, St. Paul, Minnesota, United States of America; Harvard Medical School, United States of America

## Abstract

The traditional quantitative genetics model was used as the unifying approach to derive six existing and new definitions of genomic additive and dominance relationships. The theoretical differences of these definitions were in the assumptions of equal SNP effects (equivalent to across-SNP standardization), equal SNP variances (equivalent to within-SNP standardization), and expected or sample SNP additive and dominance variances. The six definitions of genomic additive and dominance relationships on average were consistent with the pedigree relationships, but had individual genomic specificity and large variations not observed from pedigree relationships. These large variations may allow finding least related genomes even within the same family for minimizing genomic relatedness among breeding individuals. The six definitions of genomic relationships generally had similar numerical results in genomic best linear unbiased predictions of additive effects (GBLUP) and similar genomic REML (GREML) estimates of additive heritability. Predicted SNP dominance effects and GREML estimates of dominance heritability were similar within definitions assuming equal SNP effects or within definitions assuming equal SNP variance, but had differences between these two groups of definitions. We proposed a new measure of genomic inbreeding coefficient based on parental genomic co-ancestry coefficient and genomic additive correlation as a genomic approach for predicting offspring inbreeding level. This genomic inbreeding coefficient had the highest correlation with pedigree inbreeding coefficient among the four methods evaluated for calculating genomic inbreeding coefficient in a Holstein sample and a swine sample.

## Introduction

Genomic relationship among individuals is a measure of genomic relatedness or similarity among individuals and allows genomic prediction and variance component estimation to use theoretical results and computational strategies of best linear unbiased prediction (BLUP). Genomic relationship is calculated using genome-wide single nucleotide polymorphism (SNP) markers without referencing to pedigree information. Therefore, genomic relationship is particularly appealing for wildlife and animal populations where pedigree information is unavailable or incomplete.

The first attempt to use marker information to construct the **G** matrix for using BLUP to predict total marker effects was the approach of Nejati-Javaremi *et al.* [1] that defines the **G** matrix as twice the average marker IBS probability (IBS  =  identity by state). VanRaden derived the first genomic additive relationship formula based on the standardization of the 2-1-0 (or 0-1-2) coding of SNP genotypes [2]. This formula divides the 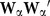 matrix by the total expected heterozygosity of all SNP markers, where 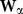 is the model matrix for SNP additive effects with each SNP coding being the deviation of the 2-1-0 coding from its mean. This approach leads to the prediction of genomic breeding values [2], [3]. Another method by Hayes and Goddard uses the average of the diagonal elements of the 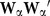 matrix in the denominator of the genomic relationships [3]. Rather than using a common denominator for all relationships, a third method divides each SNP's additive coding by the SNP's heterozygosity based on VanRaden's method for additive effects [4]. Da *et al.* proposed the quantitative genetics model that partitions a genotypic value into breeding value and dominance deviation for genomic prediction and variance component estimation of additive and dominance effects [5], [6]. The genomic additive relationships using the quantitative genetics model were identical to those based on standardization of SNP 2-1-0 coding. VanRaden derived the genomic dominance relationship formula based on the quantitative genetic model (Personal communication from P. VanRaden to Y. Da, March 3, 2013, described in [6]), and Da *et al.* [6] extended the method of Hayes and Goddard for genomic additive relationships [3] to genomic dominance relationships. Su *et al.* proposed genomic dominance relationships using the approach of standardization of the 0-1-0 dominance coding [7]. Vitezica *et al.* [8] derived the dominance relationships using the quantitative genetics model and showed that the genomic dominance relationship of Su et al. [7] was a reparameterization of the quantitative genetics model. Genomic relationships on average were consistent with the theoretical expectation of the corresponding pedigree relationships [6]. This consistency provides a justification for the interpretation of genomic relationships in parallel to pedigree relationships. In contrast to pedigree relationships, genomic relationships are realized relationships calculated from genome-wide SNP markers resulting from generations of drift, recombination, mutation and selection that are not considered by pedigree relationships, and estimates of genomic relationships for different individuals generally had their own genomic specificity unobservable from the pedigree estimates although on average genomic and pedigree relationships were consistent [6].

Inbreeding is a major issue in animal breeding and wildlife conservation because inbreeding may result in inbreeding depression associated with reduced survival and fertility [9], [10]. Inbreeding coefficients calculated from SNP markers do not require pedigree information and hence have wider applicability than pedigree inbreeding coefficients that cannot be calculated without pedigree information. Assuming the parallelism between genomic and pedigree relationships, inbreeding coefficients could be calculated using diagonal elements of the genomic additive relationship matrix as a function of the SNP model matrix for genomic prediction [11]. To improve the correlation between genomic and pedigree inbreeding coefficients, Yang *et al.* [4] proposed different diagonal elements of the genomic additive relationship matrix that are not from the SNP model matrix for genomic prediction, and Keller *et al.* [12] showed that genomic inbreeding coefficients using runs of homozygosity (ROH) achieved higher correlation with pedigree inbreeding coefficients than the method of Yang *et al*. [4]. All these methods for calculating genomic inbreeding coefficients use the individual's own SNP data. However, predicting offspring inbreeding levels before the offspring were born is an important issue in breeding plans. For this purpose, a method of calculating offspring inbreeding coefficient from parental genomic relationships can be developed.

In this study, we show that quantitative genetics theory can be the unifying theory for deriving existing and new genomic additive and dominance relationships and for studying the theoretical differences among various definitions of genomic relationships. We propose a method for calculating genomic inbreeding coefficient using a combination of genomic correlation from the quantitative genetics model, classical definition of inbreeding coefficient, and the theoretical expectation of genomic additive relationships. We also explore numerical differences among various genomic relationships and inbreeding coefficients using a Holstein sample and a swine sample.

## Methods

### Quantitative genetics model for SNP markers assuming HWE

Under the assumption of Hardy-Weinberg equilibrium (HWE), the traditional quantitative genetics model partitions a genotypic value into the summation of a common mean, breeding value and dominance deviation [6], [13], i.e.,
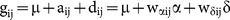
(1)where 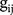  =  genotypic value of SNP genotype 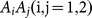, 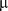  =  common mean, 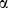  =  average effect of gene substitution, 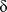  =  dominance effect, 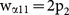, 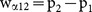, 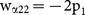, 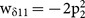, 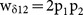, 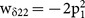, 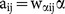  =  breeding value, 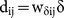  =  dominance deviation, and where 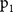  =  frequency of 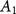 allele and 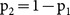  =  frequency of 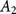. Since each individual has one genotype for each SNP, the breeding value and dominance deviation of each SNP can be re-indexed as those of individual j with SNP i. The total genomic breeding value and total genomic dominance deviation of m SNP markers of individual j are 
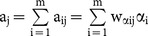
 and 
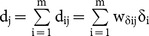
. The mathematical expectations of breeding values and dominance deviations are null under HWE, i.e., 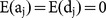. The expected additive and dominance variances of the 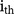 SNP based on the classical formulae from quantitative genetics [13] are:



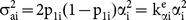
(2)

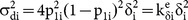
(3)where 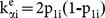 and 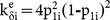. The average of expected additive and dominance variances of the m SNPs are:



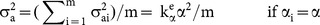
(4)

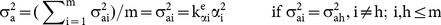
(5)

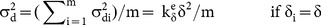
(6)

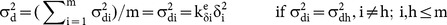
(7)where 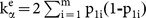 and 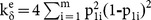.

### Genomic and pedigree covariances between two individuals

Based on the genomic breeding value and dominance deviation defined in Equation 1 and the results that the mean values of breeding values and dominance deviations are null assuming HWE [13], the covariances between genomic breeding values and between genomic dominance deviations of two individuals are:



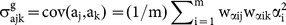
(8)

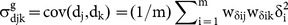
(9)


Using pedigree relationships, the covariances between breeding values and between dominance deviations of two individuals [13], [14] are:



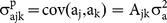
(10)

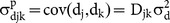
(11)where 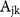  =  additive relationship between individuals j and k  =  2× (coancestry coefficient between individuals j and k), and 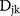  =  dominance relationship between individuals j and k.

### Mixed model for genomic prediction and variance component estimation

The mixed model to implement the quantitative genetics model of Equation 1 can be expressed as:
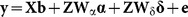
(12)where **Z**  =  N × q incidence matrix allocating phenotypic observations to each individual  =  identity matrix for one observation per individual (N  =  q), **α**  =  m ×1 column vector of gene substitution (additive) effects of SNP markers, 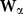  =  q × m model matrix of **α**, **δ**  =  m ×1 column vector of dominance effects of SNP markers, 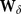  =  q × m model matrix of **δ**, **b**  =  c ×1 vector of fixed effects, and **X**  =  N × c model matrix of **b**. Assumptions for the first and second moments are: 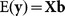, 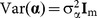, 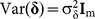, 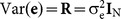, where 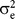  =  residual variance, **I**_m_  =  m × m identity matrix, and **I**_N_  =  N × N identity matrix. The **W** matrices of Equation 12 are the primary information for calculating genomic relationships and inbreeding coefficients.

The average of the additive or dominance SNP coding in Equation 12 can be shown to be null, i.e., 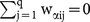 and 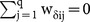 under the assumption of Hardy-Weinberg equilibrium (HWE) (S1 Text, Part A), so that the means of breeding values and dominance deviations for each SNP are null. Using this result, the sample additive and dominance variances of the 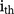 SNP are:



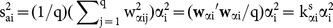
(13)

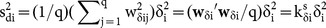
(14)where 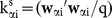, 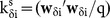, 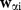  =  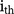
column of **W**_α_ in Equation 12 and 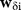  =  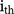 column of **W**_δ_ in Equation 12 corresponding to the 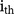 SNP. The average of sample additive and dominance variances of the m SNPs are:
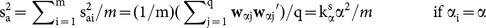
(15)

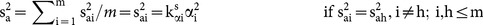
(16)

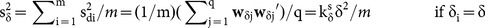
(17)

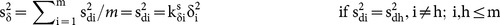
(18)where 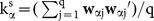, 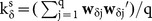, 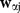  =  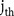 row of **W**_α_ in Equation 12, and 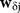  =  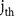
row of **W**_δ_ in Equation 12.

## Results and Discussion

### Genomic relationships derived from the quantitative genetics model

#### General formulations of genomic additive and dominance relationships

The general formulations of genomic additive and dominance relationships are obtained by equating the genomic covariances of Equations 8–9 to the pedigree covariances of Equations 10–11. Solving 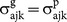 for 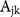
and solving 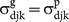 for 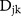 yield:



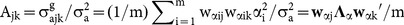
(19)

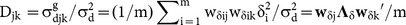
(20)where 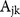  =  genomic additive relationship, 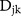  =  genomic dominance relationship, 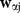 or 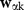  =  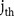
or 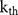 row of 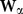 in Equation 12, 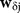 or 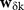  =  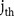 or 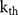 row of 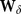 in Equation 12, 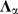  =  diag{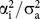} and 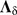  =  diag{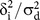}, i  =  1,..,m. In matrix notations, Equations 19–20 can be expressed as:



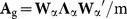
(21)

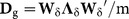
(22)where **A**_g_  =  genomic additive relationship matrix and **D**_g_  =  genomic dominance relationship matrix.

Various existing and new definitions can be derived from Equations 19–20 or 21–22. In this study, four existing definitions of genomic relationships and two new definitions are derived based on these equations (Table 1). From the point of view of the quantitative genetics model, Definitions I-III assume equal SNP effects, Definitions IV-VI assume equal SNP variances across SNP markers, Definitions I and IV use expected SNP variance, and Definitions II and V use sample SNP variance. The standardization of SNP coding for defining genomic relationships is an across-SNP standardization using a common denominator for all SNPs for Definitions I-III, and is a within-SNP standardization of each SNP using its own SNP variance for Definitions IV-VI.

**Table 1:** Six definitions of genomic additive and dominance relationships.

	Equal SNP effects	Equal SNP variances
	(Across-SNP standardization)	(Within-SNP standardization)
Expected variances	Definition I ( Equations 23 – 24 )	Definition IV ( Equations 27 – 28 )
	•Additive relationships [2]	•Additive relationships, off-diagonals are the same as in [4]
	•Dominance relationships [6]	•Dominance relationships [This article]
Sample variances	Definition II ( Equations 25 – 26 )	Definition V ( Equations 29 – 30 ) [This article]
	•Additive relationships: [3]	•Additive relationships
	•Dominance relationships [6]	•Dominance relationships
Genomic correlations	Definition III ( Equations 31 – 32 , and A_ij_ from Definition I or II) [6]	Definition VI ( Equations 31 – 32 and A_ij_ from Definition IV or V) [This article]
	•Additive correlations	•Additive correlations
	•Dominance correlations	•Dominance correlations

#### Definition I – equal SNP effects (across-SNP standardization), expected variances

Assuming equal SNP additive and dominance effects, 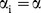 and 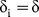, and using the expected additive and dominance variances of Equations 2–3, the genomic relationships of Equations 19–20 reduce to:



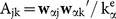
(23)

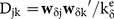
(24)where 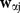 or 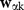  =  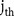 or 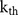
row of 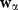 in Equation 12, 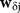 or 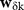  =  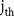 or 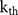 row of 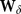 in Equation 12, 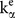 is defined in Equation 4, and 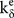 is defined in Equation 6. Equations 23–24 are the same as VanRaden's formulae [2], [6].

#### Definition II – equal SNP effects (across-SNP standardization), sample variances

Assuming equal SNP effects for all SNP markers and using the sample variances of Equations 15 and 17, the genomic relationships of Equations 19–20 reduce to:



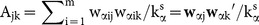
(25)

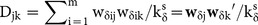
(26)where 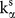 and 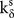 are defined in Equations 15 and 17. Equation 25 is the same as in [3], and Equation 26 is the same as in [6].

#### Definition IV – equal expected SNP variances (within-SNP standardization)

Assuming equal additive and dominance variances rather than equal additive and dominance effects across SNP markers and using the expected variances of Equations 2–3, the genomic relationships of Equations 19–20 reduce to:



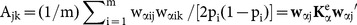
(27)

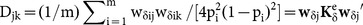
(28)where 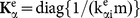 and 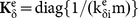, i  =  1,..,m, with 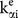 defined in Equation 2 and 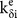 defined in Equation 3. Off-diagonal elements of Equation 27 are the same as the off-diagonal elements in [4].

#### Definition V – equal sample SNP variances (within-SNP standardization)

Using sample variances of Equations 13–14 rather than the expected variances of Equations 2–3, the genomic relationships of Equations 19–20 reduce to:



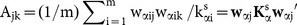
(29)

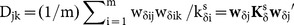
(30)where 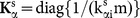 and 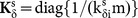, i  =  1,..,m, with 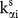 defined in Equation 13 and 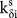 defined in Equation 14.

Definitions I-II and IV-V of genomic relationships can be represented by two sets of formulations in matrix notations. For Definitions I and II assuming equal SNP effects, 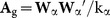 and 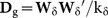, where 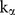  =  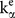 defined in Equation 4 and 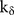  =  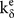 defined in Equation 6 for Definition I, or 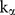  =  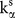
defined in Equation 15 and 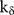  =  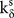 defined in Equation 17 for Definition II. For Definitions IV and V assuming equal SNP variances, 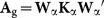 and 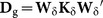, with 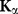  =  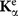 defined in Equation 27 and 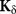  =  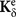
defined in Equation 28 for Definition IV, or 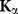  =  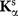 defined in Equation 29 and 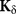  =  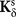 defined in Equation 30 for Definition V.

#### Genomic correlations

Genomic relationships between two individuals of Definitions I-II and IV-V as off-diagonal elements of the relationship matrices are not mathematically comparable for genomic relatedness because those off-diagonal elements are not exactly correlations. Genomic correlations (Definitions III and VI) among different individuals are mathematically comparable and provide a reference to evaluate other genomic relationship definitions.

#### Definitions III and VI – genomic correlations

The general formulations of genomic additive and dominance correlations are:



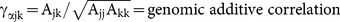
(31)

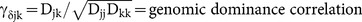
(32)where 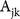 and 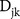 can be from any of Definitions I, II, IV and V. Equation 31 is the genomic version of Wright's coefficient of relationship or theoretical correlation between relatives [13], [15]. Based on Equations 31–32, the genomic additive correlation matrix (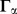) and the genomic dominance correlation matrix (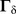) can be expressed as:



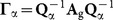
(33)

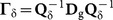
(34)where 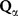  =  diag{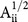}  =  m × m diagonal matrix, and 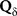  =  diag{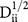}  =  m × m matrix. The diagonal elements of these two correlation matrices are ‘1’. The off-diagonal elements of 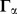 are 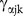 and the off-diagonal elements of 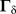 are 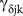 between individuals (j ≠ k). We will refer to Equations 31–34 as ‘Definition III’ if 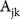 and 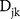 are from Definition I or II (Equations 23–24 or 25–26), or as ‘Definition VI’ if 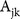 and 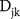 are from Definition IV or V (Equations 27–28 or 29–30). It can be readily shown that 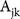 and 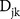 values from Definitions I and II yield identical 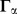 and 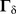 of Definition III. Numerical results showed that 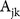 and 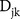 values from Definition IV and V were nearly completely correlated (correlation  =  0.999, S1 Table). Therefore, we will use 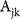 and 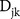 values from Definition V only in Definition VI. The six definitions of genomic additive and dominance relationships are implemented in GVCBLUP 3.9 [16], which is freely available at http://animalgene.umn.edu.

#### Genomic inbreeding coefficient based on parental genomic additive relationship

We formulate genomic inbreeding coefficients using parental genomic relationships based on the following results: 1) The inbreeding coefficient of an individual is the coancestry coefficient between the parents, 2) 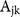  =  additive relationship between individuals j and k  =  2×(coancestry coefficient between individuals j and k) [13], and 3) Genomic additive relationships on average are consistent with pedigree ‘2×(coancestry coefficient)’ according to results in this article and in our previous report [6]. With these results, we define the genomic inbreeding coefficient of an individual as:



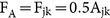
(35)or
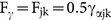
(36)where individuals j and k are the parents of the individual; 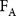  =  genomic inbreeding coefficient using parental additive genomic relationship of Definition I, II, IV or V; 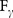  =  genomic inbreeding coefficient using parental additive genomic correlation of Definition III or VI; 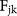  =  genomic coancestry coefficient between parents j and k; 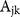  =  genomic additive relationship between the parents calculated from any of Definitions I, II, IV or V; and 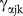  =  genomic correlation between the parents from Definition III or VI. In this study, 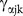 of Definition III uses 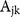, 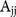 and 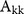 from Definition II, and 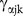 of Definition VI uses 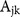, 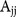 and 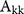 from Definition V.

### Comparison of different definitions of genomic relationships and correlations

#### Genomic additive and dominance relationships between individuals

**Figure 1:**
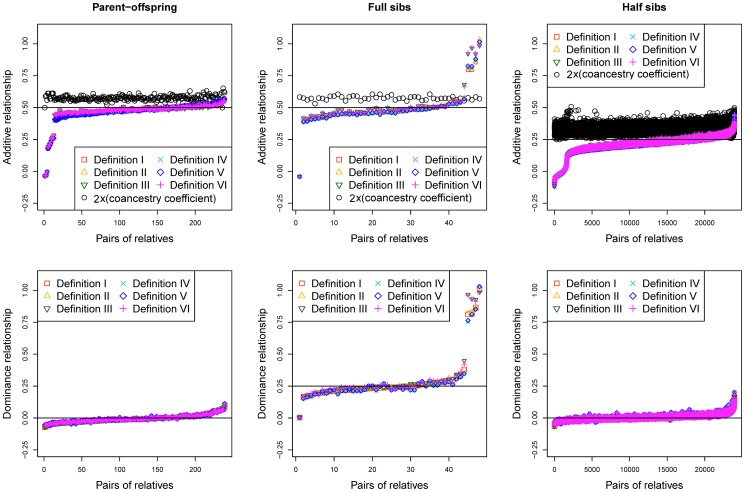
Genomic additive and dominance relationships by Definitions I-VI for parent-offspring (239 pairs), full-sibs (48 pairs) and half-sibs (23,941) of the Holstein sample.

**Figure 2:**
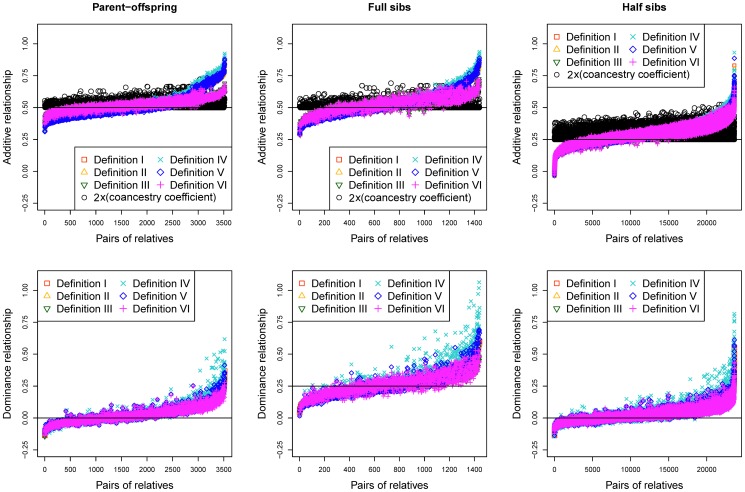
Genomic additive and dominance relationships by Definitions I-VI for parent-offspring (3518 pairs), full-sibs (1441 pairs) and half-sibs (23,628 pairs) of the swine sample.

The average values of genomic additive and dominance relationships were consistent with theoretical expectations of pedigree relationships in the Holstein sample (Fig. 1) and the swine sample (Fig. 2).

For the Holstein sample, the average genomic additive relationships were below but close to those of ‘2×(coancestry coefficient)’ calculated from the pedigree data. The genomic additive relationships of Definitions I-VI were 0.459–0.469 for parent-offspring, 0.489–0.515 for full-sibs, and 0.205–0.212 for half-sibs, compared to ‘2×(coancestry coefficient)’ average value of 0.574 for parent offspring, 0.575 for full-sibs, and 0.341 for half-sibs (Table 2).

**Table 2:** Genomic and pedigree relationships of the Holstein sample.

Definition	Additive		Dominance	
	Mean±SD	Range	Mean±SD	Range
Parent-offspring (239 pairs)				
I	0.459±0.087	−0.038, 0.574	−0.006±0.029	−0.071, 0.092
II	0.468±0.089	−0.039, 0.584	−0.006±0.029	−0.072, 0.094
III	0.469±0.084	−0.037, 0.556	−0.006±0.029	−0.073, 0.089
IV	0.461±0.088	−0.034, 0.567	−0.005±0.028	−0.057, 0.109
V	0.465±0.088	−0.035, 0.571	−0.005±0.027	−0.062, 0.113
VI	0.469±0.084	−0.035, 0.556	−0.005±0.027	−0.058, 0.107
2×(coancestry coefficient)	0.574±0.022	0.500, 0.652	-	-
Full-sibs (48 pairs)				
I	0.489±0.143	−0.042, 1.017	0.290±0.188	0.004, 1.011
II	0.498±0.146	−0.042, 1.036	0.294±0.191	0.004, 1.027
III	0.515±0.159	−0.041, 0.985	0.306±0.206	0.004, 0.986
IV	0.488±0.146	−0.042, 1.008	0.283±0.186	0.005, 1.032
V	0.491±0.147	−0.042, 1.015	0.284±0.186	0.005, 1.029
VI	0.514±0.159	−0.041, 0.984	0.307±0.204	0.005, 0.984
2×(coancestry coefficient)	0.575±0.016	0.531, 0.605	-	-
Half-sibs (23,941 pairs)				
I	0.205±0.073	−0.118, 0.436	0.003±0.020	−0.066, 0.168
II	0.208±0.074	−0.120, 0.444	0.003±0.019	−0.067, 0.171
III	0.212±0.074	−0.119, 0.438	0.003±0.021	−0.067, 0.168
IV	0.201±0.072	−0.109, 0.439	0.003±0.019	−0.058, 0.202
V	0.203±0.072	−0.110, 0.442	0.003±0.021	−0.059, 0.202
VI	0.210±0.073	−0.110, 0.439	0.004±0.020	−0.061, 0.201
2×(coancestry coefficient)	0.341±0.023	0.250, 0.508	-	-

For the swine sample, the average genomic additive relationships were almost the same as those of ‘2×(coancestry coefficient)’ calculated from the pedigree data. The genomic additive relationships of Definitions I-VI were 0.513–0.534 for parent offspring, 0.526–0.543 for full-sibs and 0.289–0.299 for half-sibs, compared to ‘2×(coancestry coefficient)’ average value of 0.528 for parent offspring, 0.530 for full-sibs, and 0.294 for half-sibs (Table 3).

**Table 3:** Genomic and pedigree relationships of the swine sample.

Definition	Additive		Dominance	
	Mean±SD	Range	Mean±SD	Range
Parent-offspring (3518 pairs)				
I	0.534±0.090	0.334, 0.856	0.033±0.066	−0.137, 0.368
II	0.517±0.087	0.324, 0.829	0.032±0.064	−0.132, 0.356
III	0.523±0.043	0.376, 0.691	0.029±0.060	−0.148, 0.300
IV	0.533±0.108	0.321, 0.924	0.038±0.084	−0.130, 0.618
V	0.513±0.099	0.310, 0.880	0.030±0.065	−0.116, 0.414
VI	0.522±0.044	0.369, 0.685	0.026±0.059	−0.139, 0.278
2×(coancestry coefficient)	0.528±0.031	0.500, 0.723	-	-
Full-sibs (1441 pairs)				
I	0.543±0.100	0.300, 0.891	0.272±0.087	0.044, 0.598
II	0.526±0.097	0.290, 0.862	0.263±0.084	0.042, 0.579
III	0.529±0.067	0.284, 0.726	0.265±0.074	0.044, 0.499
IV	0.542±0.112	0.290, 0.938	0.287±0.138	0.017, 1.063
V	0.522±0.105	0.287, 0.889	0.257±0.103	0.015, 0.691
VI	0.527±0.067	0.278, 0.719	0.262±0.081	0.016, 0.528
2×(coancestry coefficient)	0.530±0.037	0.500, 0.692	-	-
Half-sibs (23,628 pairs)				
I	0.299±0.091	−0.037, 0.830	0.028±0.049	−0.136, 0.551
II	0.289±0.088	−0.036, 0.804	0.027±0.048	−0.131, 0.533
III	0.295±0.073	−0.034, 0.660	0.026±0.047	−0.124, 0.413
IV	0.298±0.097	−0.030, 0.932	0.031±0.060	−0.147, 0.816
V	0.286±0.091	−0.034, 0.886	0.026±0.049	−0.138, 0.611
VI	0.293±0.071	−0.032, 0.662	0.025±0.048	−0.122, 0.438
2×(coancestry coefficient)	0.294±0.039	0.250, 0.525	-	-

Genomic additive relationships from the swine sample were clearly more consistent with the pedigree relationships than those from the Holstein sample. This difference likely was due to two factors. First, most Holstein cattle with SNP genotypes were distributed in the last 3–5 generations of the pedigree that approximately comprised of ten generations [17], whereas pigs with SNP data were distributed across the entire swine pedigree (S1 Figure). Consequently, the Holstein cattle with SNP genotypes on average were subjected to more generations of genetic sampling than the pigs with SNP genotypes, resulting in less genomic relationships among the Holstein cattle. Second, sources of samples could have contributed to different degrees of genomic relatedness: the Holstein sample was from a diverse source of three companies and five universities [18], whereas the swine sample was from one company only [19]. In both Holstein and swine samples, genomic correlations of Definitions III and VI had mean additive correlations that were slightly closer to the mean of pedigree additive relationships (Tables 2 and 3). This slight advantage could be the reason why Definitions III and VI had better genomic inbreeding coefficients, as to be shown. Overall, the average genomic additive relationships were remarkably consistent with the theoretical expectation of ‘2×(coancestry coefficient)’.

For genomic dominance relationships, only full-sibs are expected to have dominance relationship value of 0.25 assuming no inbreeding. Parent-offspring and half-sibs are expected to have no dominance relationship. The observed genomic dominance relationships were consistent with these theoretical expectations. The average genomic dominance relationships of Definitions I-VI were 0.283–0.306 in the Holstein sample and were 0.257–0.272 in the swine sample for full-sibs, and were nearly zero for parent-offspring and half-sibs in both samples. The higher mean values of genomic dominance relationships of full-sibs in the Holstein sample were due to the three outliers that were nearly ‘1’ among the 48 data points (Fig. 1). With the removal of those three outliers that likely were identical twins, the mean of additive relationships drops to 0.228 for Definition I to 0.237 for Definition VI, and the mean of dominance relationships drops to 0.231 for Definition V or 0.249 for Definition VI. Overall, the average genomic dominance relationships were also remarkably consistent with the theoretical expectations.

#### Realized versus expected relatedness

Genomic and pedigree relationships had a major difference: genomic relationships had individual genomic specificity and large variations not observed in pedigree relationships. For the Holstein sample, the range of genomic additive relationships was −0.039 to 0.584 for parent-offspring, compared to pedigree relationships of 0.500–0.653; −0.042 to 1.036 for full-sibs (the 1.036 value likely was due to identical twins), compared to pedigree relationships of 0.531–0.605; and −0.120 to 0.444 for half-sibs, compared to pedigree relationships of 0.250–0.508 (Table 2). For the swine sample, the range of genomic additive relationships was 0.310–0.924 for parent-offspring, compared to pedigree relationships of 0.500–0.692; 0.278–0.938 for full-sibs, compared to pedigree relationships of 0.531–0.605; and −0.030 to 0.932 for half-sibs, compared to pedigree relationships of 0.250–0.525 (Table 3). Genomic dominance relationships also had large variations (Tables 2 and 3). These large variations indicate that genomic relationships can be used to find least related genomes within the same family or among closely related individuals on the pedigree. This is important for minimizing genomic similarity in breeding plans and wildlife conservation, particularly in populations with small effective population sizes such as in endangered species. In contrast, pedigree relationships generally do not reflect real variations of genomic relatedness because genomic and pedigree relationships were mostly uncorrelated except for the case of half-sibs where the correlation between genomic and pedigree additive relationships was 0.212–0.216 for the Holstein sample and was 0.141–0.247 for the swine sample (S1 Table). These results indicate that using pedigree relationships to select individuals with least related genomes would have only limited success. Genomic relationships can be viewed as the realized genetic relationships among individuals, because each individual's genome is the realization of various factors affecting an individual's genetic composition, including genetic sampling from parents to offspring, recombination, selection and mating systems over generations, whereas pedigree relationships do not consider all these factors. Therefore, genomic relationships should be preferred to pedigree relationships for measuring genomic relatedness of different individuals.

#### Genomic inbreeding coefficients

We compared four methods for calculating genomic inbreeding coefficients: i) Using the individual's diagonal element of genomic additive relationship based on the SNP model matrix for genomic prediction: 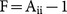, where 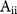  =  the 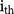 diagonal element of 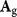 by Definition I, II, IV or V from the SNP model matrix for genomic prediction; ii) Using the individual's diagonal element of genomic additive relationship designed for calculating inbreeding coefficient (not from SNP model matrix for genomic prediction) [4], [12]: 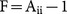, where 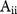  =  the 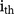
diagonal element of Definition IVb; iii) Using parental genomic additive relationship: 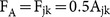 (Equation 35 using 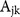 from Definition I, II, IV or V); and iv) Using parental genomic additive correction: 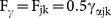 (Equation 36 using 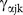 from Definition III or VI). The swine sample was used to compare these four methods (Table 4). The averages of genomic inbreeding coefficients of Methods ii)-iv) were close to the average of pedigree inbreeding coefficients, whereas the averages of genomic inbreeding coefficients Methods i) had the largest departure from the average of pedigree inbreeding coefficients. Method iv) using 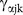 had exactly the same or almost the same largest inbreeding coefficient as that from the pedigree, whereas Methods i) and ii) all had much larger variations of inbreeding coefficients measured by SD and the range of the inbreeding coefficient estimates. Method iv) using 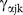 of Definition VI had the highest correlation (r) with pedigree inbreeding coefficients (r = 0.434), followed by Method iv) using 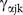 of Definition III (r = 0.419), Method iii) using 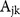 from Definitions I, II, IV and V (r = 0.367–0.380), Method ii) of Yang *et al.* [4] (r = 0.305), and Method i) using 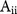 from Definitions I, II, IV and V (r = 0.071–0.131) (Table 5). While having the highest correlation with pedigree inbreeding coefficients, Method iv) as a method using parental SNP data also maintained most of the genomic specificity of the methods using the individual's own SNP data as discussed below.

**Table 4:** Statistical summary of genomic inbreeding coefficients of 1022 individuals with genotyped parents in the swine sample.

Method		Short name, source of A_ii_, A_jk_ or γ_αjk_	Mean±SD	Range
i)	F = A_ii_–1	F-I, A_ii_ of Equation 23	0.013±0.098	−0.156, 0.436
	F = A_ii_–1	F-II, A_ii_ of Equation 25	−0.019±0.095	−0.183, 0.390
	F = A_ii_–1	F-IV, A_ii_ of Equation 27	0.009±0.132	−0.197, 0.541
	F = A_ii_–1	F-V, A_ii_ of Equation 29	−0.026±0.120	−0.220, 0.478
ii)	F = A_ii_–1	F-IVb, [4], [12]	0.024±0.056	−0.098, 0.309
iii)	F_A_ = 0.5A_jk_	F_A_-I, A_jk_ of Equation 23	0.026±0.048	−0.060, 0.190
	F_A_ = 0.5A_jk_	F_A_ –II, A_jk_ of Equation 25	0.025±0.046	−0.058, 0.184
	F_A_ = 0.5A_jk_	F_A_ –IV, A_jk_ of Equation 27	0.028±0.048	−0.062, 0.199
	F_A_ = 0.5A_jk_	F_A_ –V, A_jk_ of Equation 29	0.025±0.045	−0.058, 0.188
iv)	F_γ_ = 0.5γ_αjk_	F_γ_-III, γ_αjk_ from Equations 33 and 25	0.024±0.043	−0.061, 0.159
	F_γ_ = 0.5γ_αjk_	F_γ_-VI, γ_αjk_ from Equations 33 and 29	0.023±0.042	−0.059, 0.154
Pedigree	F_p_	-	0.021±0.024	0.000, 0.159

Definition IVb: The individual's diagonal element of genomic additive relationship of each SNP is 1/p_2_, 0 and 1/p_1_ for 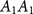, 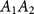 and 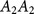 genotypes respectively, where p_1_  =  allele frequency of *A*_1_, and p_2_ = 1−p_1_ [4], [12]. F_p_ is the pedigree inbreeding coefficient.

**Table 5:** Correlation (r) between genomic and pedigree inbreeding coefficients.

	F-II	F-IV	F-V	F-IVb	F_A_-I	F_A_-II	F_A_-IV	F_A_-V	F_γ_-III	F_γ_-VI	F_p_
F-I	1.000	0.986	0.988	0.867	0.806	0.806	0.811	0.804	0.768	0.753	0.131
F-II		0.986	0.988	0.867	0.806	0.806	0.811	0.804	0.768	0.753	0.131
F-IV			0.999	0.790	0.763	0.763	0.772	0.763	0.717	0.700	0.071
F-V				0.794	0.761	0.761	0.769	0.761	0.716	0.700	0.080
F-IVb					0.834	0.834	0.837	0.835	0.823	0.820	0.305
F_A_-I						1.000	0.997	0.998	0.993	0.988	0.377
F_A_-II							0.997	0.998	0.993	0.988	0.377
F_A_-IV								0.999	0.987	0.997	0.367
F_A_-V									0.990	0.985	0.380
F_γ_-III										0.989	0.419
F_γ_-VI											0.434

F-I, F-II, F-IV, F-V, F-IVb, F_A_-I, F_A_-II, F_A_-IV, F_A_-V, F_γ_-III and F_γ_-VI are defined in Table 4. F_p_ is the pedigree inbreeding coefficient.

**Figure 3:**
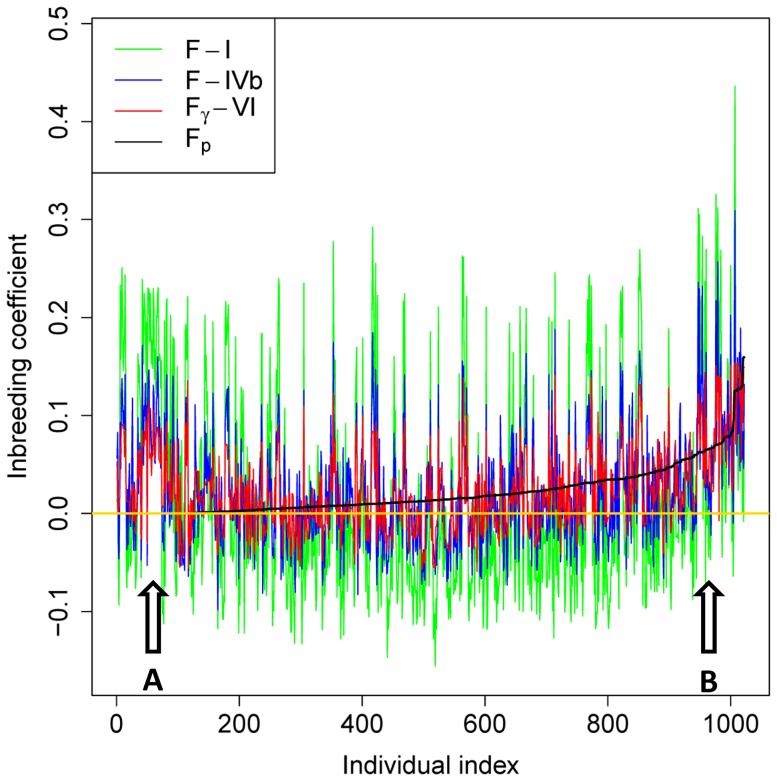
Comparison of three methods for calculating genomic inbreeding coefficients with pedigree inbreeding coefficients using a swine sample of 1022 individuals with genotyped parents. The three representative genomic methods for estimating inbreeding coefficient mostly had similar patterns of deviations from the pedigree estimates, but F-I (Method i) had the largest variations and F_γ_-VI (Method iv) had the smallest variations. For the group of individuals marked by ‘A’, all three genomic methods had high inbreeding coefficients whereas the pedigree method had ‘0’ inbreeding coefficients. For the group of individuals marked ‘B’, all three genomic methods had lower inbreeding coefficients than the pedigree estimates that were among the highest inbreeding coefficients in this sample.

The individual-by-individual comparison of Methods i), ii) and iv) with the pedigree method showed that Method iv) had similar patterns of the deviations as Method i) and ii) from the pedigree estimates of inbreeding coefficients and maintained most of the individual genomic specificity of a realized inbreeding coefficient. Fig. 3 shows that the three representative genomic methods for estimating inbreeding coefficient mostly had similar patterns of deviations from the pedigree estimates, but F-I (Method i) had the largest variations and F_γ_-VI (Method iv) had the smallest variations. For the group of individuals marked by ‘A’, all three genomic methods had high inbreeding coefficients whereas the pedigree method had ‘0’ inbreeding coefficients. These large differences between the genomic and pedigree estimates could have been due to pedigree errors or missing pedigree information. If either problem were true, this would be an example showing the usefulness of genomic inbreeding in the absence of pedigree information or in the presence of pedigree errors. For the group of individuals marked ‘B’, all three genomic methods had lower inbreeding coefficients than the pedigree estimates that were among the highest inbreeding coefficients in this sample. The correlation of inbreeding coefficients was 0.70–0.75 between Method iv) and Method i), and was 0.82 between Method iv) and Method ii), indicating that Method iv) maintained most of the genomic specificity of Methods i) and ii) while having the highest correlation with pedigree inbreeding coefficients (Table 5). Method iv) and the other methods (including the ROH method to be discussed) could be complementary methods for calculating genomic inbreeding coefficients: Method iv) for predicting offspring inbreeding level using parental SNP genotypes even before the offspring were born, and the other methods for calculating genomic inbreeding coefficient using the individual's own SNP genotypes. Method iv) could be a useful genomic tool for managing inbreeding levels in breeding plans and wildlife conservation by calculating the predicted inbreeding coefficients for all hypothetical offspring of all possible mates and by selecting the mates with the lowest predicted offspring inbreeding coefficients, similar to the approach of a software for minimizing inbreeding in breeding plans using pedigree information [20].

The high correlation between genomic inbreeding coefficients from Method iv) and pedigree inbreeding coefficients likely was due to two reasons. First, genomic correlations had smaller variations than genomic relationships, SD  = 0.043–0.044 for Definitions III and VI of genomic additive correlations, and SD  = 0.087–0.108 for Definitions I, II IV and V of genomic additive relationships (Table 3). Second, Method iv) used the same theoretical formula as pedigree inbreeding coefficient, i.e., inbreeding coefficient of the individual  =  coancestry coefficient of the parents, except that Method iv) uses parental SNP genotypes and pedigree inbreeding coefficient uses parental pedigree information. Within Method iv), the substantially higher correlation of Equation 36 than Equation 35 with the pedigree approach could be due to the fact that the genomic 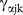 average values of Definition VI were closer to the pedigree average 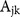 than genomic 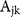 values of Definitions I, II, IV and V, as shown in Tables 2 and 3. The exact reason why genomic inbreeding coefficients using the individual's diagonal genomic additive relationship of Definitions I, II, IV and V had the lowest correlation with pedigree estimates was unclear, but two potential reasons could be identified. First, the diagonal elements of Definitions I, II, IV and V fluctuated above and below ‘1’ (S2 Figure) so that about half of the individuals had diagonal values below ‘1’. Second, diagonal elements of genomic relationships had large variations (Table 6, S2 Figure). It is worth noting that Definition IV had the largest variations in genomic relationships (additive and dominance), and its genomic inbreeding coefficient had the least correlation with pedigree inbreeding coefficient (r = 0.071, Table 5). Method ii) was a substantial improvement over Method i) in correlation with pedigree estimates, r = 0.305 for Method ii), compared to r = 0.071–0.131 for Method i). However, diagonal elements used by Method ii) also had a large number of individuals with diagonal elements less than ‘1’ (S2 Figure). The averages of diagonal elements of Definitions I, II, VI and V used by Method i) and Definition IVb used by Method ii) were about ‘1’ for the Holstein sample compared to the average of 1.05 of the pedigree diagonal values, and were 1.033–1.036 for the swine sample compared to the average of 1.017 of the pedigree diagonal values (Table 6).

**Table 6:** Statistical summary of diagonal values of additive and dominance relationships.

Definition	Additive		Dominance	
	Mean±SD	Range	Mean±SD	Range
Holstein cattle (n = 1654)				
I	0.982±0.056	0.751, 1.545	0.984±0.058	0.791, 1.631
II	1.000±0.057	0.764, 1.573	1.000±0.059	0.804, 1.658
IV	0.983±0.066	0.776, 1.699	0.980±0.163	0.701, 3.104
V	0.991±0.066	0.783, 1.707	0.991±0.157	0.708, 3.031
IVb	0.993±0.042	0.874, 1.441	-	-
2×(coancestry coefficient)	1.050±0.021	1.000, 1.300	-	-
Swine (n = 3534)				
I	1.033±0.105	0.844, 1.649	1.034±0.123	0.825, 1.852
II	1.000±0.102	0.817, 1.600	1.000±0.119	0.798, 1.791
IV	1.035±0.142	0.803, 1.924	1.106±0.327	0.676, 3.922
V	0.999±0.131	0.780, 1.850	0.999±0.219	0.661, 3.137
IVb	1.036±0.060	0.883, 1.427	-	-
2×(coancestry coefficient)	1.017±0.027	1.000, 1.259	-	-

We were unable to make direct comparison between Method iv) and the approach of runs of homozygosity (ROH) for calculating genomic inbreeding coefficients [12], because the Holstein sample has a small number of parents with SNP genotypes and the swine sample has anonymous SNP markers with unknown chromosome position. However, based on results in this study and in Keller *et al.* [12], Method iv) would be competitive for calculating genomic inbreeding coefficients in terms of correlation with pedigree inbreeding coefficients. In Fig. 6 of Keller *et al.*, the correlation between the genomic inbreeding coefficients using ROH and the pedigree inbreeding coefficients were higher than that of Yang *et al.* [4] (or Method ii) in this article) by less than 0.10, whereas such correlation from Method iv) was 0.114–0.129 higher than Method ii) of Yang *et al.* [4].

#### GBLUP and GREML from different definitions of genomic relationships

Definitions I-VI essentially had the same GBLUP of breeding values and dominance deviations that mostly had correlations of 0.99 among GBLUP from different definitions (S2 Table). For GREML estimates of additive and dominance heritabilities, the six definitions also had similar results except the dominance heritability of Trait 3 for which Definitions IV and V had higher estimates, 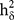  =  0.103 for Definition IV and 0.094 for Definition V, compared to 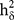  =  0.031–0.064 for the other four definitions (Table 7). The different GREML estimates of dominance heritabilities by Definitions IV and V could be due to differences between SNP dominance effects from these two definitions and those from the other definitions, see below.

**Table 7:** Estimated genomic heritabilities from the swine sample.

Trait	Heritability	Definition of genomic relationship or correlation					
		I	II	III	IV	V	VI
1	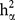	0.032	0.033	0.035	0.034	0.035	0.036
	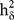	7.66×10^−7^	7.62×10^−7^	9.92×10^−7^	6.06×10^−7^	1.20×10^−6^	1.03×10^−6^
	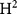	0.032	0.033	0.035	0.034	0.035	0.036
2	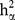	0.263	0.269	0.269	0.266	0.273	0.274
	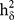	0.016	0.016	0.022	0.004	0.008	0.018
	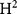	0.279	0.285	0.291	0.270	0.280	0.292
3	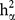	0.211	0.216	0.209	0.216	0.222	0.217
	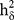	0.064	0.065	0.031	0.103	0.094	0.033
	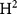	0.275	0.281	0.241	0.318	0.317	0.251
4	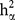	0.346	0.353	0.353	0.348	0.355	0.354
	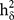	0.007	0.007	0.009	2.09×10^−7^	0.003	0.010
	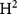	0.353	0.360	0.363	0.348	0.357	0.364
5	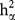	0.375	0.382	0.388	0.377	0.384	0.392
	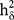	0.054	0.055	0.059	0.043	0.054	0.062
	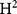	0.429	0.437	0.446	0.420	0.438	0.454

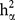
  =  additive heritability, 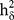  =  dominance heritability, and 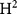 =  total heritability (or heritability in the broad sense).

#### SNP additive and dominance effects

**Figure 4:**
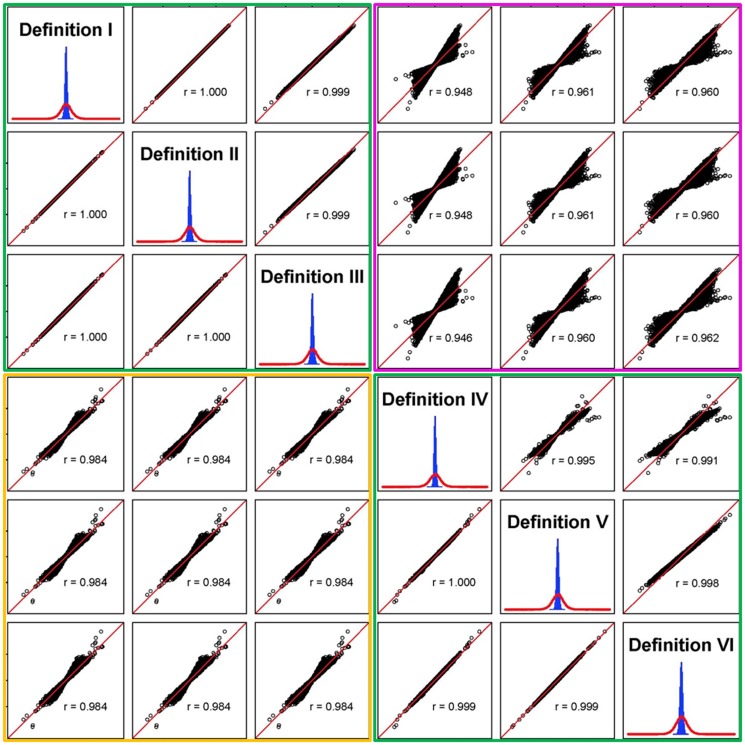
Distribution of SNP additive and dominance effects from Definitions I-VI and QQ plots of SNP effects relative to Definition I. All graphs of this figure are based on Trait 5 with 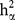  = 0.375–0.392 and 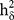  = 0.043–0.062 (Table 5). Definitions I-III in the upper green box assume equal SNP effects or across-SNP standardization, and Definitions IV-VI in the lower green box assume equal SNP variance or within-SNP standardization. Diagonal graphs are distributions of SNP additive effects (red curves) and SNP dominance effects (blue histograms). Off-diagonal graphs are QQ plots relative to Definition I, with r  =  correlation coefficient in each graph. The lower off-diagonal graphs of QQ plots of SNP additive effects, and the upper off-diagonal graphs are the QQ plots of SNP dominance effects.

Fig. 4 shows the distribution of SNP additive and dominance effects from Definitions I-VI and correlations of SNP effects between different definitions of genomic relationships. Both additive and dominance SNP effects had bell-shape distributions, but dominance effects (blue histograms in diagonal graphs) had a much narrower distribution than additive effects (red curves in diagonal graphs), consistent with the low dominance heritability and high additive heritability of the trait. By the sizes and distributions of SNP additive and dominance effects, the six definitions could be divided into two groups: Definitions I-III under the assumption of ‘equal SNP effects’ or ‘across-SNP standardization’ as one group, and Definitions IV-VI under the assumption of ‘equal SNP variance’ or ‘within-SNP standardization’ as the other group. Graphs in the upper green box showed virtually identical additive and dominance effects from Definitions I-III. The lower green box showed virtually identical additive effects from Definitions IV-VI, and somewhat different dominance effects between Definition IV and Definitions V-VI. Graphs in the gold box showed similar additive effects between Definitions I-III and IV-VI although Definition VI led to substantially better estimates of genomic inbreeding coefficients (Tables 4 and 5, Fig. 3), and graphs in the pink box showed that the differences between Definitions I-III and IV-VI were mainly in the highest and lowest dominance effects. The use of ‘expected SNP variance’ (Definitions I and IV) or ‘sample SNP variance’ (Definitions II and V) had similar SNP additive and dominance effects (gold and pink boxes).

## Conclusions

The traditional quantitative genetics model was shown to be a unifying model to derive genomic additive and dominance relationships and genomic inbreeding coefficients. Genomic additive and dominance relationships between individuals on average agreed well with the pedigree relationship, but genomic relationships were realized genetic relationships with individual genomic specificity and had large variations not observed from pedigree relationships. Genomic relationships assuming equal SNP variances had larger variations than assuming equal SNP effects. Genomic inbreeding coefficients calculated from parental genomic correlations had high correlations with pedigree inbreeding coefficients and could be an effective genomic tool for predicting offspring inbreeding levels in breeding plans.

## Supporting Information

S1 Figure **Pedigree of the swine sample with 3534 individuals.**
(PDF)

S2 Figure **Diagonal elements of genomic additive and dominance relationships and pedigree additive relationships of the swine sample with 3534 individuals.** Definition IVb of diagonal elements of additive relationships was that of Yang et al. [4]. Pedigree inbreeding coefficient was calculated by Pedigraph 2.4 [21].
(PNG)

S1 Table Correlations between genomic additive and dominance relationships under Definitions I-IV and pedigree additive relationship measured by ‘2×(coancestry coefficient)’.
(PDF)

S2 Table Correlations of breeding values, dominance deviations and total genetic values under different genomic relationship definitions.
(PDF)

S1 Text **Proofs and animal data.**
(PDF)
