# Supplementary figures and images for: Quantitative Genetics Model as the Unifying Model for Defining Genomic Relationship and Inbreeding Coefficient
Source: PLoS One. 2014 Dec 17;9(12):e114484. doi: 10.1371/journal.pone.0114484 (PMC4269408; doi:10.1371/journal.pone.0114484)

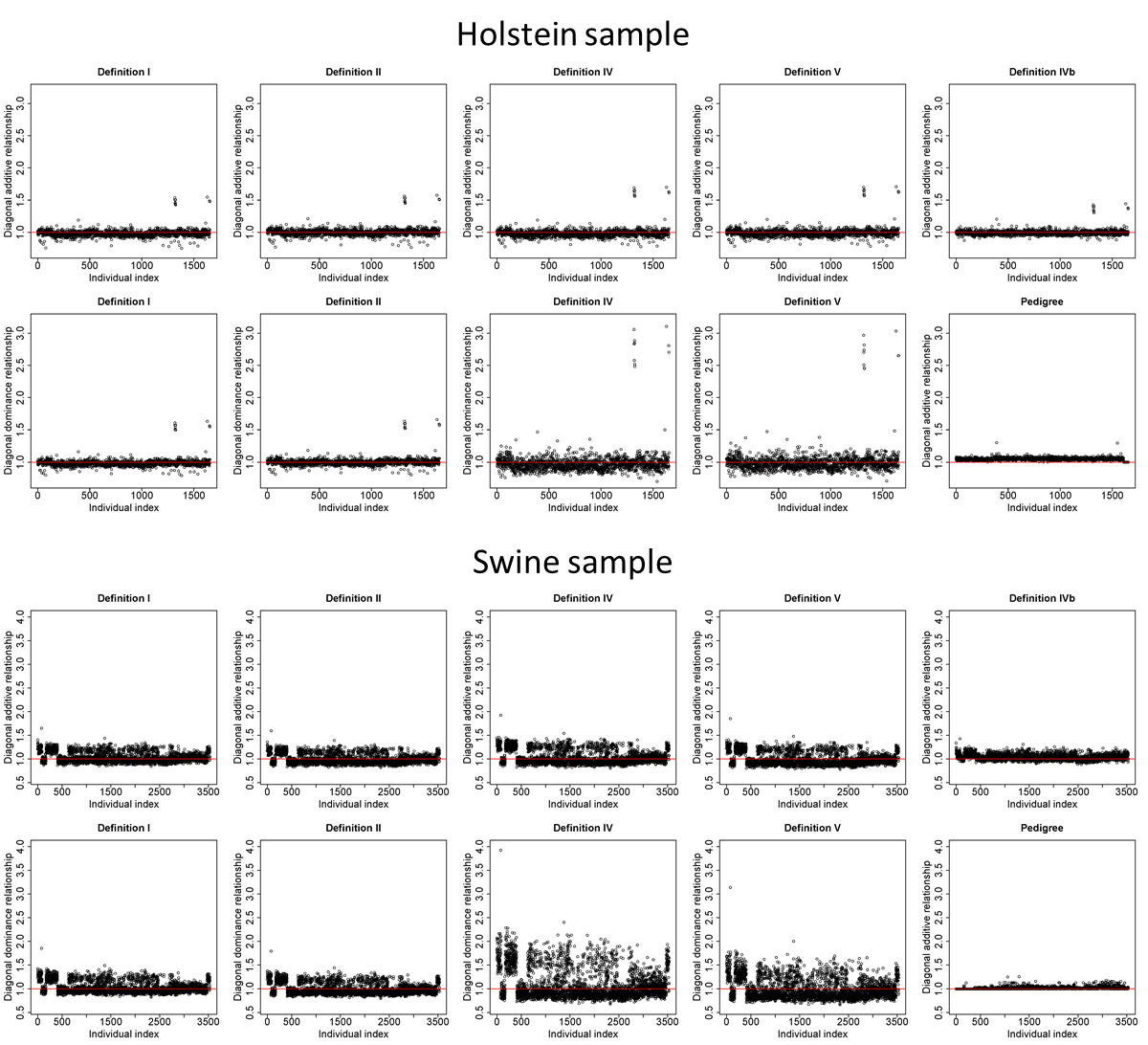

Supplement: S2 Figure — Diagonal elements of genomic additive and dominance relationships and pedigree additive relationships of the swine sample with 3534 individuals. Definition IVb of diagonal elements of additive relationships was that of Yang et al. [4]. Pedigree inbreeding coefficient was calculated by Pedigraph 2.4 [21]. (PNG) [file pone.0114484.s002.png]
