# Supplementary material for: Quantitative Genetics Model as the Unifying Model for Defining Genomic Relationship and Inbreeding Coefficient
Source: PLoS One. 2014 Dec 17;9(12):e114484. doi: 10.1371/journal.pone.0114484 (PMC4269408; doi:10.1371/journal.pone.0114484)
Supplement: S1 Table — Correlations between genomic additive and dominance relationships under Definitions I-IV and pedigree additive relationship measured by ‘2×(coancestry coefficient)’. (PDF) [file pone.0114484.s003.pdf]

Table S1. Correlations between genomic additive and dominance relationships under Definitions I-IV and pedigree additive relationship measured by '2×(coancestry coefficient)'

A) The Holstein sample

Definition	I	II	III	IV	V	VI	2×(coancestry coefficient)
Parent-offspring (239 pairs)							
I	-	1.000	0.987	0.998	0.998	0.986	0.014
II	1.000	-	0.987	0.998	0.998	0.986	0.014
III	0.9996	0.9996	-	0.984	0.984	0.999	0.017
IV	0.964	0.964	0.962	-	0.999993	0.984	-0.006
V	0.964	0.964	0.962	0.997	-	0.984	-0.006
VI	0.967	0.967	0.966	0.995	0.998	-	0.013
Full-sibs (48 pairs)							
I	-	1.000	0.981	0.999	0.999	0.981	-0.078
II	1.000	-	0.981	0.999	0.999	0.981	-0.078
III	0.993	0.993	-	0.984	0.984	0.9996	-0.089
IV	0.995	0.995	0.983	-	0.999996	0.985	-0.082
V	0.996	0.996	0.984	0.9997	-	0.985	-0.082
VI	0.992	0.992	0.998	0.986	0.988	-	-0.083
Half-sibs (23,941 pairs)							
I	-	1.000	0.996	0.998	0.998	0.996	0.215
II	1.000	-	0.996	0.998	0.998	0.996	0.215
III	0.9996	0.9996	-	0.993	0.994	0.999	0.212
IV	0.957	0.957	0.955	-	0.99999	0.995	0.213
V	0.957	0.957	0.956	0.998	-	0.995	0.213
VI	0.959	0.959	0.960	0.995	0.998	-	0.216

Upper off-diagonals: correlations between genomic additive relationships and between genomic and pedigree relationships; lower off-diagonals: correlations between genomic dominance relationships.

B) The swine sample

Definition	I	II	III	IV	V	VI	2×(coancestry coefficient)
Parent-offspring (3518 pairs)							
I	-	1.000	0.922	0.989	0.990	0.896	0.073
II	1.000	-	0.922	0.989	0.990	0.896	0.073
III	0.991	0.991	-	0.877	0.884	0.987	0.207
IV	0.930	0.930	0.897	-	0.9995	0.862	0.019
V	0.962	0.962	0.945	0.981	-	0.871	0.028
VI	0.948	0.948	0.961	0.928	0.975	-	0.204
Full-sibs (1441 pairs)							
I	-	1.000	0.882	0.989	0.991	0.865	0.073
II	1.000	-	0.882	0.989	0.991	0.865	0.073
III	0.935	0.935	-	0.827	0.840	0.995	0.111
IV	0.898	0.898	0.739	-	0.9993	0.815	0.033
V	0.946	0.946	0.829	0.978	-	0.829	0.043
VI	0.888	0.888	0.955	0.776	0.861	-	0.114
Half-sibs (23,628 pairs)							
I	-	1.000	0.962	0.992	0.994	0.952	0.168
II	1.000	-	0.962	0.992	0.994	0.952	0.168
III	0.991	0.991	-	0.933	0.942	0.995	0.247
IV	0.915	0.915	0.880	-	0.9994	0.929	0.130
V	0.955	0.955	0.936	0.981	-	0.939	0.141
VI	0.948	0.948	0.959	0.928	0.974	-	0.246

Upper off-diagonals: correlations between genomic additive relationships and between genomic and pedigree relationships; lower off-diagonals: correlations between genomic dominance relationships.
