# Supplementary material for: Quantitative Genetics Model as the Unifying Model for Defining Genomic Relationship and Inbreeding Coefficient
Source: PLoS One. 2014 Dec 17;9(12):e114484. doi: 10.1371/journal.pone.0114484 (PMC4269408; doi:10.1371/journal.pone.0114484)
Supplement: S2 Table — Correlations of breeding values, dominance deviations and total genetic values under different genomic relationship definitions. (PDF) [file pone.0114484.s004.pdf]

Table S2 Correlations of breeding values, dominance deviations and total genetic values under different genomic relationship definitions for Traits 3 and 5 of the swine sample

Trait		Definition	II	III	IV	V	VI
3	Additive	I	1.000	0.999	0.997	0.998	0.997
		II		0.999	0.997	0.998	0.997
		III			0.995	0.996	0.998
		IV				0.9995	0.997
		V					0.998
	Dominance	I	1.000	0.993	0.953	0.972	0.972
		II		0.993	0.953	0.972	0.972
		III			0.926	0.953	0.974
		IV				0.993	0.963
		V					0.982
	Genotypic	I	1.000	0.994	0.991	0.995	0.993
		II		0.994	0.991	0.995	0.993
		III			0.974	0.981	0.998
		IV				0.999	0.979
		V					0.986
5	Additive	I	1.000	0.999	0.998	0.998	0.998
		II		0.999	0.998	0.998	0.998
		III			0.998	0.998	0.998
		IV				0.99992	0.999
		V					0.999
	Dominance	I	1.000	0.997	0.965	0.979	0.977
		II		0.997	0.965	0.979	0.977
		III			0.957	0.974	0.981
		IV				0.995	0.980
		V					0.990
	Total	I	1.000	0.999	0.998	0.999	0.998
		II		0.999	0.998	0.999	0.998
		III			0.996	0.997	0.999
		IV				0.9998	0.998
		V					0.999
