# Supplementary material for: Quantitative Genetics Model as the Unifying Model for Defining Genomic Relationship and Inbreeding Coefficient
Source: PLoS One. 2014 Dec 17;9(12):e114484. doi: 10.1371/journal.pone.0114484 (PMC4269408; doi:10.1371/journal.pone.0114484)

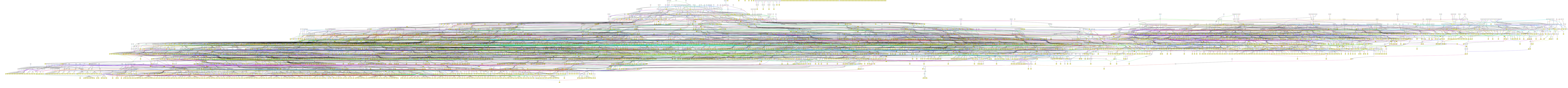

Supplement: S1 Figure — Pedigree of the swine sample with 3534 individuals. (PDF) [file pone.0114484.s001.pdf]
