# Supplementary material for: Quantitative Genetics Model as the Unifying Model for Defining Genomic Relationship and Inbreeding Coefficient
Source: PLoS One. 2014 Dec 17;9(12):e114484. doi: 10.1371/journal.pone.0114484 (PMC4269408; doi:10.1371/journal.pone.0114484)
Supplement: S1 Text — Proofs and animal data. (PDF) [file pone.0114484.s005.pdf]

Quantitative genetics model as the unifying model for defining genomic relationship and inbreeding coefficient

Chunkao Wang and Yang Da*
Department of Animal Science, University of Minnesota, St. Paul, MN 55108, USA
*Corresponding address: Yang Da, Email: yda@umn.edu

Text S1: Proofs and animal data

Part A: Sample means of breeding values and dominance deviations

The partition of a genotypic value into breeding value and dominance deviation assuming Hardy-Weinberg equilibrium (HWE) [1,2] can be summarized by Table A1.

Table A1. Breeding value and dominance deviation

Genotype	A_1A_1	A_1A_2	A_2A_2
Number of individuals	N_{11}	N_{12}	N_{22}
Genotypic frequency: general expression	$P_{11} = N_{11}/N$	$P_{12} = N_{12}/N$	$P_{22} = N_{22}/N$
Genotypic frequency: under HWE	p^2	2pq	q^2
Breeding value	$a_{11} = 2q\alpha$	$a_{12} = (q-p)\alpha$	$a_{22} = -2p\alpha$
SNP additive coding	2q = 2–2p	q–p = 1–2p	–2p = 0–2p
Dominance deviation	$d_{11} = -2q^2\delta$	$d_{12} = 2pq\delta$	$d_{22} = -2p^2\delta$
SNP dominance coding	$-2q^2$	2pq	$-2p^2$

α = the average effect of gene substitution, δ = dominance effect.

The sample means of breeding values and dominance deviations for the i_{th} SNP are: $\bar{a}_i = (\sum_{j=1}^{q} w_{\alpha ij}/N)\alpha_i$ and $\bar{d}_i = (\sum_{j=1}^{q} w_{\delta ij}/N)\delta_i$. To prove these sample means to be null is to prove $\sum_{j=1}^{q} w_{\alpha ij}/N = 0$ and $\sum_{j=1}^{q} w_{\delta ij}/N = 0$. The mean of additive SNP coding is:

$$\sum_{j=1}^{q} w_{\alpha ij}/N = P_{11}\times(-2q) + P_{12}\times(p-q) + P_{22}\times 2p = (0-2q)P_{11} + (1-2q)P_{12} + (2-2q)P_{22}$$
$$= (P_{12} + 2P_{22}) - 2q(P_{11} + P_{12} + P_{22}) = 2q - 2q = 0$$

where $q = P_{22} + \frac{1}{2}P_{12} = 1 - p = 1 - (P_{11} + \frac{1}{2}P_{12})$. The mean of the dominance SNP coding is:

$$\sum_{j=1}^{q} w_{\delta ij}/N = P_{11}\times(-2q^2)+P_{12}\times 2pq+P_{22}\times(-2p^2)$$

$$=-2P_{11}(P_{22}+\frac{1}{2}P_{12})^2+2P_{12}(P_{11}+\frac{1}{2}P_{12})(P_{22}+\frac{1}{2}P_{12})-2P_{22}(P_{11}+\frac{1}{2}P_{12})^2$$

$$=-2P_{11}(P_{22}^2+P_{12}P_{22}+\frac{1}{4}P_{12}^2)+2P_{12}(P_{11}P_{22}+\frac{1}{2}P_{11}P_{12}+\frac{1}{2}P_{12}P_{22}+\frac{1}{4}P_{12}^2)-2P_{22}(P_{11}^2+P_{11}P_{12}+\frac{1}{4}P_{12}^2)$$

$$=2(-P_{11}P_{22}^2-P_{11}P_{12}P_{22}+P_{11}P_{12}P_{22}-P_{11}^2P_{22}-P_{11}P_{12}P_{22})-\frac{1}{2}(P_{11}P_{12}^2+P_{12}^2P_{22})+\frac{1}{2}P_{12}^3+(P_{11}P_{12}^2+P_{12}^2P_{22})$$

$$=-2P_{11}P_{22}(P_{22}+P_{12}-P_{12}+P_{11}+P_{12})+\frac{1}{2}P_{12}^2(P_{11}+P_{12}+P_{22})$$

$$=\frac{1}{2}P_{12}^2-2P_{11}P_{22}$$

Under HWE, the above mean of dominance SNP coding reduces to zero.

Part B: Cattle and swine data for comparing genomic relationships and inbreeding coefficients

A dairy cattle sample with 1654 Holstein cows [3] was used to compare different definitions of genomic relationships. After SNP selection based on MAF (minor allele frequency) > 0.05, 41,550 SNP markers remained for analyses. The Holstein pedigree had about ten generations with genotyped cows in the last 3-5 generations of the pedigree [4]. A publically available swine genomics dataset from PIC nucleus pig line with anonymous genome-wide SNP markers and phenotypes [5] was also used to compare various genomic relationships and inbreeding coefficients. This sample included 3534 animals with Illumina PorcineSNP60 SNP data and five anonymous phenotypes. The swine pedigree had about 20 generations with genotyped pigs dispersed over almost all generations. A total of 45,376 SNP markers remained for analyses after filtering with MAF > 0.05, and proportion of missing SNP genotypes < 0.10. The pedigree of the swine sample (Figure S1) and the pedigree coancestry and inbreeding coefficients were produced using Pedigraph 2.4 [6].

References

1. Falconer DS, Mackay TFC (1996) Introduction to Quantitative Genetics. Harlow, Essex, UK: Longmans Green.
2. Da Y, Wang C, Wang S, Hu G (2014) Mixed model methods for genomic prediction and variance component estimation of additive and dominance effects using SNP markers. PloS One 9: e87666.
3. Cole JB, Wiggans GR, Ma L, Sonstegard TS, Lawlor TJ, et al. (2011) Genome-wide association analysis of thirty one production, health, reproduction and body conformation traits in contemporary US Holstein cows. BMC Genomics 12: 408.

4. Ma L, Wiggans G, Wang S, Sonstegard T, Yang J, et al. (2012) Effect of sample stratification on dairy GWAS results. BMC Genomics 13: 536.
5. Cleveland MA, Hickey JM, Forni S (2012) A common dataset for genomic analysis of livestock populations. G3: Genes| Genomes| Genetics 2: 429-435.
6. Garbe JR, Da Y P (2008) Pedigraph: A software tool for the graphing and analysis of large complex pedigree. User manual Version 2.4. Department of Animal Science, University of Minnesota. http://animalgene.umn.edu.
